# Hospital physician payment mechanisms in Austria: do they provide gateways to institutional corruption?

**DOI:** 10.1186/s13561-017-0148-4

**Published:** 2017-03-01

**Authors:** Margit Sommersguter-Reichmann, Adolf Stepan

**Affiliations:** 1 Department of Finance, Karl-Franzens University Graz, Resowi G2, A-8010 Graz, Austria; 2 Institute of Management Science, Technical University Vienna, Theresianumgasse 27, A-1040 Vienna, Austria

## Abstract

Institutional corruption in the health care sector has gained considerable attention during recent years, as it acknowledges the fact that service providers who are acting in accordance with the institutional and environmental settings can nevertheless undermine a health care system’s purposes as a result of the (financial) conflicts of interest to which the service providers are exposed. The present analysis aims to contribute to the examination of institutional corruption in the health sector by analyzing whether the current payment mechanism of separately remunerating salaried hospital physicians for treating supplementary insured patients in public hospitals, in combination with the public hospital physician’s possibility of taking up dual practice as a self-employed physician with a private practice and/or as an attending physician in private hospitals, has the potential to undermine the primary purposes of the Austrian public health care system. Based on the analysis of the institutional design of the Austrian public hospital sector, legal provisions and directives have been identified, which have the potential to promote conduct on the part of the public hospital physician that systematically undermines the achievement of the Austrian public health system’s primary purposes.

## Background

Analyses regarding the interplay between hospitals’ and physicians’ financial incentives and the quantity and quality of health care supply and expenditure have a long history. The relevant contributions can be divided broadly into two major streams: first there are those that concentrate on the direct link between different payment systems and service provision in terms of the volume and range of services, technology use and service quality, and second there are contributions that choose a broader perspective by taking into account the conflicts of interest that the service providers face in light of the legal and institutional settings.

The impact that conflicts of interest may exert on the conduct of health care professionals has been investigated in the medical literature since the 1980s [1], followed by an intensive examination by social scientists, (health) economists, lawyers and ethicists. According to Rodwin [2], p. 9, physicians are confronted with conflicts of interest whenever ‘[…] their interests […] compromise their independent judgment […].’ Thompson [3], p. 137, clarified a conflict of interest as ‘[…] a set of circumstances that are reasonably believed to create a substantial risk that professional judgment of a primary interest will be unduly influenced by a secondary interest.’ There is a broad consensus that the primary interest of physicians should be to act in the best interests of the patient, particularly as the relationship between physician and patient is often considered to be fiduciary [4]. Physicians’ secondary interests comprise, among others, professional or scientific recognition, continuing professional education and specialization, as well as financial gain. Koelewijn et al. [5] investigated various personal interests of hospital physicians in the Netherlands, which ranged from helping patients as well as possible – supposedly the primary interest – to having a say in work and to earning a good income. Among the ten major interests under investigation, helping patients in the best possible way ranked first while earning a good income was the fifth most important interest. Jegers et al. [6] analyzed remuneration per service (so-called fee-for-service, FFS), per day, per case (diagnoses-related group, DRG), per patient (capitation) and per period (global budget) and concluded that service providers do indeed respond to financial incentives, so – depending on the type of financial incentive – effects on the volume, range, frequency and continuity of services, number of hospitalizations, length of stay (LOS) and (self-) referrals can be observed. Similar results can be found in the relevant literature [7–16], also considering the effects on quality and risk selection [17–20].

The broader literature stream that problematizes the fact that a physician who is acting in accordance with the legal and institutional settings can nevertheless undermine a system’s purposes as a result of the (financial) conflicts of interest to which he or she is exposed dates back to the early work on congressional ethics by Thompson, who identified conduct on behalf of congressional members, which ‘[…] under certain conditions is a necessary or even desirable part of institutional duties […]’ and ‘[…] has a tendency to damage the legislature of the democratic process’ [21], p. 7. More generally speaking, this phenomenon describes a situation in which the institutional setting produces incentives that create conflicts of interest and subsequently promote behavior on the part of those who perform the duties within the institution that systemically compromises the institution’s purposes. Thompson [21] termed this phenomenon institutional corruption. Lessig [22], p. 553, further elaborated that institutional corruption exists ‘[…] when there is a systemic and strategic influence which is legal, or even currently ethical, that undermines the institution’s effectiveness by diverting it from its purpose or weakening its ability to achieve its purpose […].’ Currently, a lively discussion about the ‘ultimate’ definition of institutional corruption is ongoing [21, 23–25] and so far has resulted in a range of definitional approaches. Marks [26], p. 11, however, noted that a broader perspective regarding institutional corruption may be useful because ‘[…] one definition works better in some contexts and not so well in others.’ For this contribution, a symbiosis of Thompson’s and Lessig’s definitions of institutional corruption is used as we see institutional corruption as a situation in which the institutional design produces incentives for those who perform the duties within the institution that create conflicts of interest and subsequently promote behavior that systemically and strategically influences the institution’s effectiveness by diverting it from its purposes.

Systemic dependencies and informal practices in health care that have the potential to distort a health care system’s societal mission through institutional corruption have only occasionally been investigated in the relevant literature. Most studies have focused on the pharmaceutical industry [27–30] and clinical research [31–33], as well as medical care provision in general [34–37]. Rodwin [27] highlighted the influence of the pharmaceutical industry on medical research, publications and key stakeholders. Gagnon [28] problematized the misalignment of the pharmaceutical industry’s financial interests with the purposes of the health care system, which allows institutional corruption to breed, while Light et al. [29] provided evidence that, during the last decades, pharmaceutical firms have mainly developed minor-variation new drugs instead of investing in clinically superior new drugs following several systemic dependencies, such as inadequate monitoring by and commercialization of the Food and Drug Administration. Whitaker and Cosgrove [30] investigated the influence of the pharmaceutical industry on the American Psychiatry Association. Wilmshurst [31] emphasized the key role of medical schools and universities in setting standards for clinical research, and Redman [32] drew attention to the research ethics in biomedical research. Emanuel and Steiner [33] investigated institutional conflicts of interest and potential remedies in the context of clinical research. Ensor [34] analyzed ‘endemic’ corruption in the form of informal payments for health care services in transition economies. Informal payments coincide – at least to a certain extent – with institutional corruption, as they are ‘[…] part of daily life to the point at which it is no longer considered illegitimate’ [34], p. 244. García-Prado and González [35] examined the consequences of dual practice for health care in terms of access, efficiency and quality because the complexity of the financial incentives and the possible conflicts of interest are likely to increase when physicians work at the same time in the public and the private sectors. They concluded that a statement on the net effects of dual practice is difficult to make as a result of the limited empirical evidence on these issues. Socha and Bech [36] predominantly found arguments supporting the negative effects of dual practice, among which were an increased focus on and effort in private practice at the expense of public health care provision resulting from the higher income in private practice, an increase in public waiting lists to stimulate the demand for equivalent private services, the redirection of profitable patients from public waiting lists to private practice, the overprovision of health services in the public sector to obtain a good reputation for treating patients in the private sector and the use of public resources for privately offered medical services. In relation to dual practice, Ferrinho et al. [37] identified financial and other barriers to access to health services resulting from the predatory behavior of clinicians who generate additional demand for their own privately offered services, the absenteeism of clinicians in public institutions due to the competition for time, the emergence of (financial) conflicts of interest, which are supposed to result in poor quality of publicly provided services, the outflow of public resources for providing services in private practice and the corruption in the health care sector.

While combating individual corruption, that is, illegal individual behavior, has a long history in health care (see, e.g., [34, 38–40], the analysis of institutional corruption has only recently become the focus of scientific research. As reasons for the neglect of institutional corruption, Thompson [24], p. 19, listed the following: 1) institutional corruption is closely related to conduct, which is part of the job, 2) there is a common perception that no solution to this problem exists, 3) there is predominantly a concentration on personal misconduct rather than institutional failures on the part of the public and the media and 4) individual corruption still dominates.

The current analysis is intended to contribute to the examination of institutional corruption in the health sector by investigating whether the Austrian health care system is prone to institutional corruption. In particular, we analyze whether the current payment mechanism of remunerating salaried public hospital physicians separately for treating supplementary-insured patients in public hospitals, in combination with public hospital physicians’ possibility of taking up dual practice as self-employed physician with private practice and/or as attending physician in private hospitals, provides a systemic and strategic influence that undermines the health care system by diverting it from its purposes [23]. Following the theory of institutional corruption, we first identify the primary purposes of the Austrian public health care system. Next, we scrutinize the institutional design regarding special fees and dual practice in the public hospital sector to identify provisions that potentially promote behavior on the part of the public hospital physician that may systematically undermine the primary purposes of the Austrian public health care system.

The research question is motivated as follows. Special fees are an important source of income for public hospital physicians; they are legitimate but may introduce (financial) conflicts of interest on the part of public hospital physicians, so there may be a substantial risk that their financial interests will weaken the ability to achieve the ascribed purposes of the Austrian health care system. The diversion from the primary purpose(s) of the health care system may even be aggravated by the public hospital physician’s opportunity to take up dual practice as a self-employed physician with private practice and/or as attending physician in private hospitals as a result of the possible conflicts of interest the physician may face when providing services in different settings in which different remuneration systems are likely.

We focus on special fees in combination with the above-mentioned dual practice options, because we suspect several adverse effects on health care expenditure and patient care (e.g. two-tier medicine and/or inefficiency in service provision), which are the final result of a failure of the institutional design rather than individual misconduct. Undoubtedly, many more manifestations of corrupt behavior, such as bribery, procurement corruption, improper marketing relations, misuse of high level positions, undue reimbursement claims and fraud and embezzlement [38] – whether in the form of individual corruption, institutional corruption or a combination of the two – are worth analyzing. Covering all potential manifestations of corrupt behavior, however, is beyond the scope of this paper. Given a loss to corruption estimated to range between €1.1 and €3.6 billion in Austria [41], the identification of potential gateways to corruption, however, should be a priority issue.

The remaining part of the paper is structured as follows. In Section 2 the characteristics of the Austrian public hospital sector and the institutional design approach of Oliveira [42], which has been chosen to investigate potential gateways to institutional corruption in the Austrian health care system, are presented. The primary purposes, institutional design and potential gateways to institutional corruption resulting from the payment of special fees to public hospital physicians in combination with the above mentioned dual practice options in Austria are introduced and discussed in Section 3. The paper concludes with a short summary, discusses the shortcomings and provides prospects for future research.

## Methods

### The Austrian public hospital sector

The Austrian health care system is based on the principles of solidarity, universal, equal and low-threshold access to health services, high quality and efficiency in service delivery [43]. The legally specified responsibilities for the health sector are split between the federal government, the state governments and the statutory health insurance (SHI) funds: while the federal government is responsible for the framework legislation, the implementation and enforcement of regulations are the responsibility of the state governments, which are also in charge of the relevant hospital sector. The self-governing SHI funds have been delegated the competencies for health care services provided outside hospitals. The split of competencies has resulted in a highly fragmented, complex and partly opaque health care system with regard to the delivery and financing of health services.

The Austrian hospital population can be classified according to different (partly overlapping) structural features, such as the care sector (acute, non-acute), care type (general, specialized), care level (standard, extended, maximum, specialized), hospital type (general, specialized), type of financing (DRG-based, not DRG-based), legal status (with/without public law status), benefit status (not-for-profit, for-profit) and ownership type (public, private). According to the Austrian Federal Hospitals Act (KAKuG), the legal status is decisive for the distinction between public and private hospitals: Public hospitals are general and specialized acute care hospitals and nursing facilities for chronically ill patients which are granted public law status. Public law status, which requires the hospital, among others, to be run on a not-for-profit basis (irrespective of the type of ownership), imposes additional obligations (e.g. to charge only officially fixed tariffs) on the hospital in exchange for additional rights (e.g. the right to subsidies in the case of deficits) [44]. In 2017 113 hospitals (41% of all hospitals) operated under public law status, providing around 67% of the overall hospital bed capacity. Of these 113 public hospitals, 105 hospitals were acute care hospitals which were eligible for public financing through state health funds. The state health funds allocate a more or less fixed budget, which mainly comprises predefined percentages of sales taxes and valorized contributions of SHI funds, on a DRG basis. The remaining eight hospitals were not DRG-financed facilities for the chronically ill (mainly financed out of SHI). Of the 161 private hospitals, i.e. of the hospitals without public law status, 40 hospitals were non-profit hospitals (comprising DRG-funded and not DRG-funded facilities) and 121 hospitals were for-profit hospitals [45]. Private, for-profit hospitals (private hospitals henceforth) also receive public funds (mainly SHI contributions) through a private hospitals’ fund for services provided to socially insured patients that are covered by the SHI scheme.

Public (and private non-profit) hospitals may be authorized through state legislation to run a special class in addition to the general class and to approve further fees, so-called special fees, for the treatment in the special class [46]. The special class is supposed to cover increased patient demands regarding accommodation (one- or two-bed room) and food as well as free choice of the employed public hospital physician (mostly senior doctors or department heads). Each of the nine Austrian states has made use of the right to authorize a special class in public hospitals and to approve additional charges in the form of special fees.

The special fees are usually covered through private hospital cost insurance (also termed supplementary health insurance, as medical care as such is publicly funded, irrespective of whether it is provided in the general or the special class). In 2015 around 36% of the Austrian population had taken out some form of supplementary health insurance. Approximately 56% of these supplementary-insured Austrians were covered through private hospital cost insurance [47]. In 2014 the expenditures of supplementary health insurance on hospitals amounted to 7% of the public expenditure on hospitals [48].

Operating a special class in public hospitals has frequently been justified as follows. Salaries in the public health care sector are perceived as low. Special fees, which comprise – among others – a hospital and a physician fee, are supposed to ensure the commitment of highly qualified medical staff to the public sector [49]. This ‘one-size-fits-all’ justification, however, is problematic, because the share of special fees and therefore the compensating effect regarding the salary vary considerably across hospitals, disciplines and qualification levels [50].

Public hospital services are supplied by health professionals, ranging from hospital physicians to nurses and to lab assistants. Except for university hospitals, which carry out research and teaching tasks in addition to providing public health services and in which employees of medical universities also provide public services, hospital physicians are employed by the respective hospital owner, which is in most cases a hospital company owned by the state or the municipality (public ownership) or a religious order (private ownership). Public hospital physicians receive a salary and an additional variable income, comprising, among others, special fees for the treatment of supplementary-insured patients in the special class. A study in 2014 revealed the following average shares of the yearly income of a public hospital physician: basic salary (42%), allowances, such as length-of-service or hardship allowance (12%), overtime compensation (17%), outpatient fees (8%) and physician fees (21%). The actual shares, however, may differ considerably, depending – among others – on the age and the medical field of the physician [50].

Public hospital physicians may be authorized by their employer to perform dual practice as a self-employed physician with a private practice and/or as an attending physician in private hospitals. The type and extent of dual practice of employed hospital physicians may vary considerably across states, hospitals and hospital physicians, depending, among others, on how dual practice is regulated. The dual practice of employees of medical universities, for example, is regulated by federal law (the Civil Servants Act (BDG)) or collective and operating agreements, while the dual practice of employees of privately owned public hospitals is regulated in the respective employment contract.

Supplementary-insured patients have several options to consult a senior public hospital physician: directly in his/her private practice (if additionally self-employed), indirectly via the outpatient department of the public hospital, directly in the private hospital (if he/she is also an attending physician) or indirectly through referral by another physician. The fact that public hospital physicians are allowed to offer medical services in different settings, in which they are most likely to be remunerated differently, may have an impact on medical decisions in terms of the volume, range, frequency and timing of medical services. It can also affect the setting in which specific services are offered.

Rarely available state-specific data regarding the income of chief public hospital physicians have revealed that the size of the special fees is considerable (Table 1) and even increases over time. Average income data have shown the considerable size of chief hospital physicians’ financial gain from special fees, as they are able to increase their income by more than double. The system of special fees may therefore introduce considerable conflicts of interest, as hospital physicians are ‘rewarded’ for treating supplementary-insured patients. As public hospitals benefit from special fees as well, strategic alliances may emerge.

**Table 1:** Average income components

State	Year	Average gross income in thousand € (deflated to 1994 prices)	
		Salary	Special fee
Styria [63]	1994	75.7	61.7
Styria [59]	2003	91.9 (79.2)	91.4 (78.7)
Upper Austria [50]	2014	104.0 (71.5)	156.0 (107.2)

### The institutional design approach

**Fig. 1:**
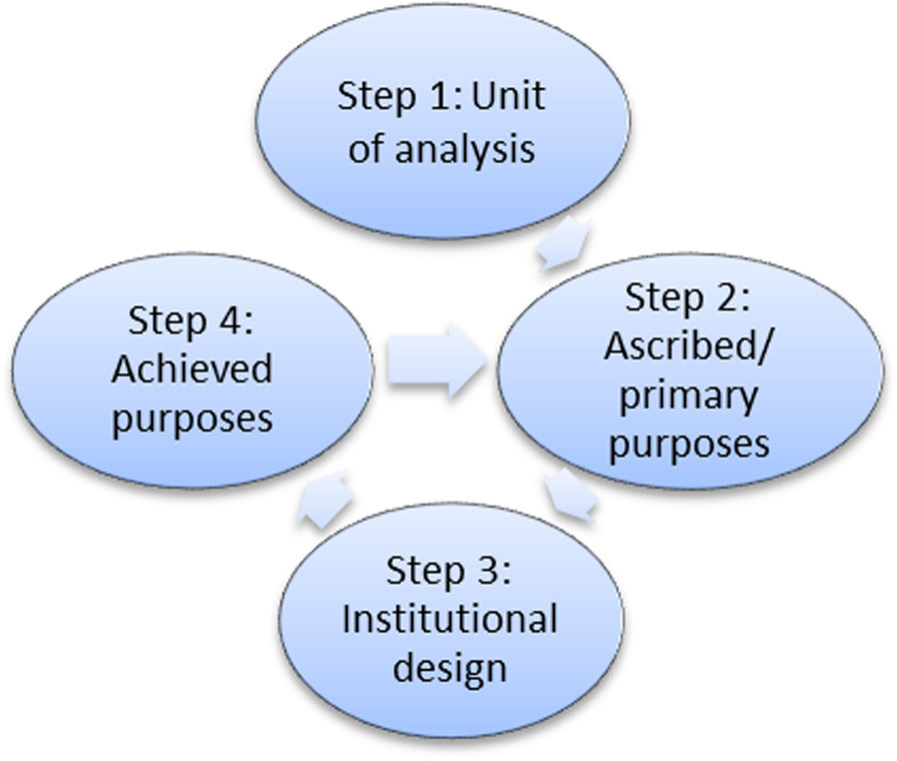
Operationalizing institutional corruption

Oliveira [42] suggested a stepwise procedure (Fig. 1) to investigate institutional corruption. In the first step, the units under analysis have to be determined. Next, the ascribed purposes of these units need to be identified. The constituent elements of an institution are frequently described in terms of vision or mission statements, while implementation is frequently asserted through bylaws and strategy plans. These implementation issues are addressed in the third step, which analyzes the institutional design in terms of the breakdown and motivation structure. This step follows a typical procedure in practice in which regular reviews of implementation strategies take place to identify any intentional or unintentional deviation from the primary purposes. These review practices are important, because they help to disclose whether groups (e.g. staff categories) within or outside the institution (e.g. lobbies) are pursuing goals other than the primary purposes without disclosing their behavior to the primary target group of the institution. The assessment of actually achieved or presumably resulting goals, given the institutional design, is the subject of the fourth step. If there is a discrepancy between the ascribed purposes and the actually achieved or presumably resulting goals, statements can be made regarding the existence of (gateways to) institutional corruption. The approach proposed by Oliveira therefore illustrates that, apart from identifying institutional corruption, it is important to be able to trace how, that is, through which channels, institutional corruption has developed, and whether it is the result of inappropriate rule making, probably influenced by external advisory bodies, or rule-gaming decisions by-passing the existing rules [51].

In our case the focus lies on public hospitals and public hospital physicians in particular. It has to be considered, however, that these service providers are embedded within a hierarchical legal system, in which the ascribed purposes of public hospitals are defined at the level of the constitutional law. The framework legislation, that is, federal laws, may also outline mission statements, which are tantamount to primary purposes. The institutional design in terms of the breakdown and motivation structure at the public hospital level is heavily defined by state laws and regulations, because the states are responsible for hospital care. The states are therefore supposed to implement the primary purposes outlined in the framework legislation at the federal level in appropriate goals at the state and, thus, public hospital level. If the institutional design promotes behavior that actually or presumably leads to a deviation from the primary purposes, institutional corruption and potential gateways to institutional corruption are identified.

The evaluation of outcomes, however, can be based on various approaches. First, if relevant data are available, an empirical analysis of them is obvious to assess the actual outcome. Alternatively, relevant knowledge from the scientific literature can be used to draw conclusions on possible outcomes in light of the breakdown and motivation structure. The latter approach is chosen for this contribution due to a lack of access to relevant data and missing data for Austria.

## Results and discussion

### Units of analysis, primary purposes and institutional design

The examination of the Austrian health care system for institutional corruption started in the first step with the definition of the units of analysis, which comprise public acute care hospitals (public hospitals henceforth) as defined in section 2.a and public hospital physicians. Since there are differences regarding the design of the regulations concerning the special fees across the nine states and the presentation of the nine state laws is beyond the scope of this paper, the provisions for a single state (Styria) are outlined representatively.

As regards the Austrian hospital sector, we identified as primary purposes the goals for the overall health care system outlined in the preambles of Austrian laws and related resolutions. The preamble of the 15a Agreement ‘Health Care Organization and Financing’ outlines that the parties (i.e. the federal and the state governments) commit themselves to comprehensive medical care for all people, regardless of age and income, based on the principles of solidarity, equal access to health services and high quality and efficiency in service delivery [43]. In 2011 the Federal Health Commission and the Austrian Council of Ministers initiated the formulation of overall health targets for Austria. Finally, 10 health targets, which mainly aim to increase the years of healthy life instead of only responding to illnesses, were officially approved following a target-setting process involving all the relevant stakeholders. Targets 2 and 10 are directly related to the major principles of the Austrian health policy as outlined in the 15a Agreement ‘Health Care Organization and Financing’: target 2 is to promote fair and equal opportunities in health, irrespective of gender, socio-economic group, ethnic origin and age, and target 10 aims to secure sustainable and efficient health care services of high quality for all [52]. In its preamble the 15a Agreement ‘Target Control Health’ [53] emphasizes the focus on these guiding principles for the Austrian health policy and public health principles. Based on these laws and the resolution regarding the Austrian health targets, we identified ‘good health,’ ‘equity’ and ‘efficiency’ as the primary purposes of the Austrian health care system, and we assume that they are also valid for the service providers who operate within the public health care system. ‘Good health’ is a typically intrinsic goal, while ‘equity’ and ‘efficiency’ are instrumental goals, meaning that, if achieved, they prepare the way for achieving ‘good health.’

These universal statements need to be translated into explicit directives so that they can be used as a baseline for service providers. We therefore first examined the concrete formulations at the federal level that set up the framework for the respective provisions regarding the special fees at the state level. Table 2 summarizes the provisions of the federal and state laws, regulations and treaties that put the primary purposes into explicit terms, that are considered potentially to exert an impact (positively or negatively) on one or more of the primary purposes and that are, to a certain extent, related to the issue of special fees. Assignments to primary purposes are made in accordance with what we considered to be the main purpose(s) of the relevant provision.

**Table 2:** Directives outlined in federal and state laws

Federal laws	Good health	Equity	Efficiency	State laws, regulations and treaties
15a Agreement ‘Target Control Health’				State treaty ‘Target Control Health’ [64]
Art. 5 (1–2) Provision of medical care at the ‘best point of service’			+	Definition of public service obligations for all care levels (primary, hospital outpatient and inpatient care) and launching implementation
Art. 5 (2–3) Coordination of services across all sectors, offering of patient-oriented and needs-based services and prevention or reduction of parallel structures	+	+	+	Development of interdisciplinary forms of care in the ambulatory setting
Art. 5 (3–2) Relief of the inpatient sector in hospitals through medically and economically justified relocation of services to day care and ambulatory care		+	+	
Art. 5 (3–7) Development of remuneration schemes, which guarantee service provision at the best point of service			+	Participation at the federal level in developing incentives to promote outpatient care
Federal Hospitals Act (KAKuG)				Styrian Hospitals Act (StKAG)
§16 Non-profit hospitals must				§51 adopts §16 KAKuG
• admit everyone in need of medical care in accordance with the hospital’s facilities	+	+		
• accommodate every patient as long as medically required	+	+	+	
• ensure that medical treatment, irrespective of accommodation in the special class, is based on the medical condition of the patient solely […]	+	+	+	
• secure that the staff, notwithstanding §27 (4) and §46 (1), must not be remunerated by patients or their family members		+/−	+/−	
• not run more than 25% of the total beds as special-class beds		+/−	+/−	
§22 (2): Public hospitals are obliged to admit any socially insured patients	+	+		§87 (1) adopts §22 (2) KAKuG
§27 (4-1) State legislation has to determine				
• whether and		+/−	+/−	§66 regulates the prerequisites for implementation of the special class
• what further charges may be levied in the special class		+/−	+/−	§75 regulates special fees
§46 (1) The department heads of university hospitals may agree a separate fee with special-class patients […], irrespective of the special-class patients’ obligations to pay special fees […], if these patients request treatment by the department head. This separate fee is not subject to §27 (4) […].		+/−	+/−	

Regarding the purpose of ‘good health’, public hospitals have to admit anyone in need of medical care, have to treat a patient as long as medically necessary and have to decide on the medical treatment based on medical grounds solely. Some of these provisions can also be related to the purpose of ‘equity,’ particularly the fundamental principles that no one in need of care must be rejected and no discrimination must be made according to the insurance status of the patient. ‘Efficiency’ is of considerable importance, because compliance with efficiency secures the financial stability of the health care system. Efficiency-related provisions are found in the 15a Agreement ‘Target Control Health,’ which emphasizes the importance of treatment at the best point of service, that is, cost-effective and efficient service provision that guarantees high-quality medical and nursing care at the right time and in the right place, the importance of coordination between different service providers, the relief of the cost-intensive Austrian inpatient sector and the development of remuneration systems that are compatible with the Austrian health targets.

For certain provisions, however, it is a priori unclear whether a positive or a negative effect on the primary purposes dominates. These include, inter alia, the prohibition of extra payments of hospital staff, which per se promotes equity and efficiency efforts. As the respective provision excludes special fees and separate fees (§46), which chief university physicians may require in addition to the possibility of charging special fees pursuant to §§16 and 27 (4) of the KAKuG, the primary purposes of equity and efficiency may be undermined considerably. Similar arguments are valid for the restriction of the number of special-class beds to 25% of the overall bed capacity: on the one hand, special-class beds cannot be increased arbitrarily; on the other hand, a 25% limit may increase the overall bed capacity or hinder the capacity-reducing efforts. With regard to the implementation of the special class in general, a positive effect in terms of generating additional revenue is prevalent; a negative effect is expected from additionally remunerating hospital physicians for providing medical care in the special class, which, in turn, must not be different from the medical care provided in the general class.

The legal provisions, which regulate the hospital and the physician fees of the state-owned public hospitals in Styria (and which provide about 91% of the public hospital bed capacity in Styria [45]) are complex and outlined in Table 3. Public hospitals are allowed to run a special class if there is a sufficient (absolute) number of beds in the general class. If accommodation in the special class is requested, hospital and physician fees apply. The hospital fee is levied to compensate for the extra costs for material and personnel in the special class and is made up of different compensation systems, ranging from a percentage of the relevant DRG fee to flat fees. The hospital owner retains the entire hospital fee. Although there shall not be any difference in medical treatment between the general class and the special class, a physician fee applies if the patient requests medical treatment in the special class. As differences in medical care are prohibited by law, there has been a lively discussion in the scientific literature what exactly the supplementary-insured patient receives in return for the physician fee [54]. Both the hospital and the physicians benefit from the physician fee. The public hospital currently receives a hospital share totaling 19%, while the physicians involved in the treatment of special-class patients receive the remaining 81% (physician share). The physician share is then divided between the affiliated physicians. The allocation procedure, which is governed by regulation and, thus, obligatory for the employees of the public state-owned hospitals, follows a complex scoring method that takes the hierarchical position, seniority and special qualifications of the physicians into account [55].

**Table 3:** Regulations regarding special fees in Styria

Special fees (StKAG)			
Public hospitals may run a special class in addition to the general class […] if the hospital provides a sufficient number of beds in the general class, particularly for those who cannot be denied hospital care. […] There shall not be any difference in medical care. The special class has to meet higher demands in terms of food and accommodation. (§66) Special fees comprise the hospital fee, the physician fee and the midwife fee in the special class and the outpatient fee.^a^ (§76)			
Outpatient care and financing	Inpatient care and financing		
Lump-sum compensation (socially insured) Special fees (self-payers only)	DRG/case fees (general class; socially insured) DRG/case fees and special fees (special class; supplementary-insured)		
	Special fees comprise [65]		
	Hospital fee	Physician fee	
	− compensation for extra costs (material, personnel) in the special class − tariffication predominantly based on flat fees and fees as a percentage of the DRG fees	− compensation for medical care provided by the department head and affiliated physicians in the special class − tariffication predominantly based on specific fees (for surgical interventions, diagnostic imaging, radiotherapy treatments), fee/day (conservative treatments), surcharges and flat fees (e.g. lab tests)	
		decomposes into [66]	
	Hospital share 100%	Hospital share 19%	Physician share 81%

^a^Midwife and outpatient fees are no longer considered

The breakdown and motivation structure with regard to the special class provides financial incentives for hospitals and hospital physicians to treat special-class patients. The question therefore arises of whether the service providers have the opportunity to boost their income through special fees, that is, whether hospital physicians can influence the demand for treatment in the special class. The widespread possibility of dual practice for (senior) hospital physicians paves the way to influencing the demand regarding elective services.

Usually, dual practice requires authorization from the employer and is limited in time. Regarding dual practice, there may be differences depending on the type of ownership across public hospitals: while privately owned hospitals usually regulate dual practice options individually under employment contracts (non-competition clause), the dual practice of physicians of the state-owned public hospitals (and the medical university) is subject to two different sources of law in Styria, the Civil Servants Act (BDG) and the Styrian Civil Servants and Pay Act (Stmk L-DBR). The BDG outlines that dual practice is any activity pursued outside employment and must not be performed if it hinders the physician from fulfilling his official duties, causes the presumption of partiality or endangers other essential interests of the service. The civil servant (i.e. the hospital physician) is obliged to notify the employer about any dual practice. The Stmk L-DBR adopts these provisions and further states that the employed physician may pursue a medical activity in another hospital or may use hospital equipment and staff only if authorized by the employer. These regulations offer the opportunity to perform dual practice and, therefore, to influence the demand in the settings in which physicians operate.

### Gateways to institutional corruption

Rodwin [2], p. 10, pointed out in quite an early study that self-employed physicians are entrepreneurs just like any other freelancers, meaning that they sell services to generate income, while the employment of physicians typically precludes entrepreneurship. The institutional setting in the Austrian hospital sector, however, opens up a broad spectrum of opportunities for physicians employed in public hospitals to act in an entrepreneurial manner. Public hospital physicians can influence their overall income by pushing their variable income components, such as the physician fee for the treatment in the special class of the public hospital, and the income resulting from authorized dual practice as self-employed physician and/or as attending physician in private hospitals.

The employed public hospital physician can proactively influence the occupancy of the special class through the referral of supplementary-insured patients from outpatient to inpatient care, because hospital and physician fees are restricted to inpatient services. This may be attractive whenever there is void capacity in the special class. This activity can boost physician income but may impede any attempts to relieve the inpatient sector because any reduction of the total bed capacity is tantamount to income loss for both the hospital owner and the hospital physician because the special-class bed capacity is linked to total bed capacity (25% rule). In addition, the tariffication of hospital and physician fees reveals that pay per intervention and pay per day are predominantly used to remunerate hospitals and hospital physicians for providing special-class services. Following the findings of relevant scientific studies [6, 8–10, 13, 16], these payment forms are likely to increase the volume and range of services. The restriction of hospital and physician fees to inpatient care and their tariffication are likely to threaten any attempts to provide services effectively and efficiently at the best point of service.

**Fig. 2:**
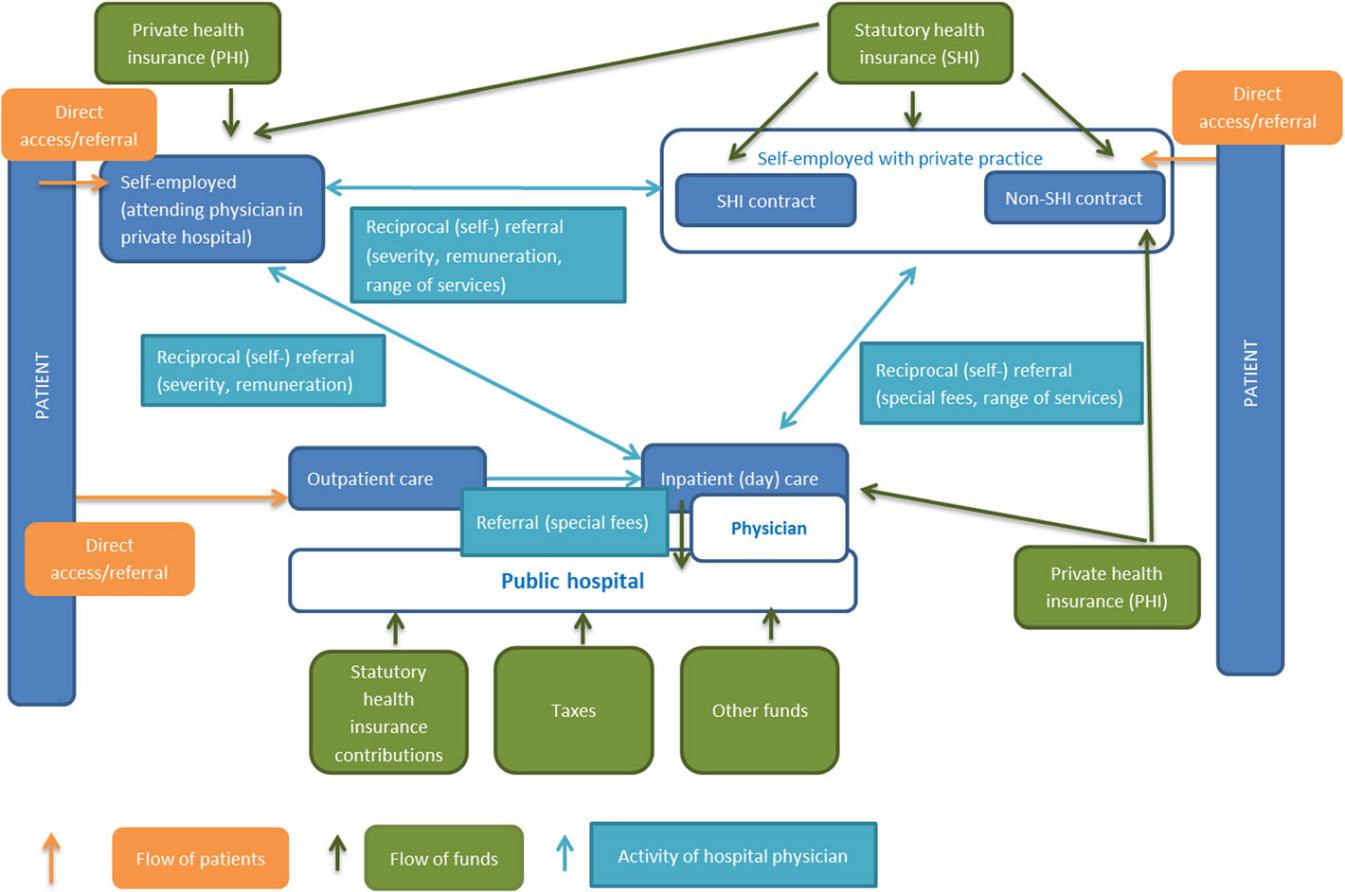
Interplay between hospital physicians’ activities in the presence of physician fees and dual practice

If any of the dual practice options outlined above is performed in addition to the employment in a public hospital, we assume that the extra work is pursued to increase the public hospital physician’s income. Although dual practice may lead to considerably adverse effects [36, 37] the institutional design of the Austrian public hospital sector permits different types of dual practice. As a consequence, intricate structures evolve regarding the flow of funds and patients and the activity that hospital physicians may undertake in the context of physician fees (Fig. 2).

A public hospital physician may be authorized to pursue dual practice as a non-contracted physician or – in rare cases – even as a contracted physician with a private practice. If a patient attends a contracted physician, the services provided are directly settled with the SHI fund (mostly on a FFS basis) that individually contracts with the respective physician. The SHI funds directly regulate the number of contracted physicians through the number of contracts offered. Patients may also attend a non-contracted physician directly. In that case they have to pay in advance and receive reimbursement to the amount of 80% of the tariff that the SHI fund would have paid for this service to a contracted physician. The SHI funds have no opportunity to regulate the supply with non-contracted physicians directly. In the context of special fees, the dual practice as a (non-)contracted physician with a private practice opens up the possibility not only of acquiring income in the form of physician fees through (self-) referral from private practice to the special class in the public hospital but also of acquiring additional income as a self-employed physician through, for example, reverse referral from the special class to private practice for medical aftercare. In Vienna, for example, referral from private practice to the special class is rewarded through ‘bringer solutions’ [56]; that is, hospital physicians are financially better off (in terms of the physician share) when they refer patients from private practice to the special class. While this may impede attempts to relieve the inpatient sector, proponents of this solution argue that it strengthens the competitiveness of the public hospital sector if the patient would otherwise have been admitted to a private hospital. This argument, however, presumes that there is no physician-induced demand and that inpatient care is the ‘best point’ for service provision. Empirical evidence on this issue is lacking in Austria. A potential approach to investigating this question could be to undertake, for a selection of comparable diagnoses, a comparison of the referral behavior of hospital physicians who run a private practice with that of contracted and non-contracted self-employed physicians without any affiliation to a hospital. This issue, however, is challenging due to the different accounting systems that apply to contracted and non-contracted physicians and the different accounting systems between the 19 SHI funds.

The analysis of the referral behavior can also be used to investigate whether the current institutional design systematically promotes a two-tier medical system: the differences in the waiting times, access, range and volume of services between socially and supplementary-insured patients, adjusted for diagnosis and age, would indicate that the current institutional design promotes behavior that undermines the system’s equity purpose. Differences in waiting times for elective treatments between socially and supplementary-insured inpatients, however, have already been disclosed [57]. Another study revealed a higher number of lab tests and a greater frequency of express lab tests for supplementary-insured patients [58]. Further sparse empirical evidence regarding the impact of supplementary health insurance on ‘good health,’ ‘equity’ and ‘efficiency’ is available through the Audit Court Reports, but the results are mainly snapshots, particularly as the findings are mostly by-products of audit assignments, the aim of which differs from the current research question. One Audit Court Report [59] revealed differences in the average LOS between socially and supplementary-insured inpatients in university hospitals in 2004 (Vienna +1.6 days, Graz +1.8 days and Innsbruck +1.5 days in the case of supplementary health insurance), which mainly occurred in orthopedic and vascular surgery departments. In 2008 the Vienna Audit Court investigated differences in assignments of elective surgeries between socially and supplementary-insured patients in the departments of orthopedics and ophthalmology, in which differences were found regarding the registration for and planning of elective surgeries [60].

Public hospital physicians may also be authorized to provide medical services as attending physicians in private hospitals. Dual practice in private hospitals is sometimes handled more restrictively (e.g. conditional on the explicit patient wish [61]), because dual practice in private hospitals competes directly with public hospitals. If dual practice as an attending physician in private hospitals is authorized and the hospital physician decides to treat a patient in the private hospital, financial resources (the hospital fee and the hospital share) are withdrawn from the public hospital. As only public hospitals are obliged to admit socially insured patients, private hospitals have a decisive strategic advantage, as they may cherry pick financially attractive patients, which, in turn, may be mirrored in the amount of remuneration that the public hospital physician receives for providing services as an attending physician in the private hospital. In the case of dual practice in private hospitals, reciprocal referral between the private and the public hospital may occur, probably depending on the severity of the illness (usually, the more severe the more appropriate the public hospital so that more complex and more expensive services then have to be provided in the public hospital) and the financial consequences for the hospital physician of treating patients in either of the two hospitals. The complexity of the referral behavior further increases if the public hospital physician works as a self-employed physician in private practice in addition to dual practice in private hospitals. Another Audit Court Report revealed that 3700 of the 32,300 employees of the Vienna Hospital Association (the main owner of public hospitals in Vienna), that is, around 11.5%, had indicated that they had engaged in dual practice in 2006. With regard to the 165 medical directors and department heads, 148, that is, 89.7%, performed dual practice [62]. Such activities may seriously impair attempts to coordinate services efficiently and effectively in all sectors and to prevent parallel structures. Further negative effects, such as increases in public waiting lists, may result from the potential absenteeism of department heads in public hospitals as a consequence of providing services in private hospitals as outlined in the relevant literature [37]. Previous reports have revealed that chief physicians had the opportunity to perform services outside the hospital starting in the early afternoon due to highly flexible work time regulation and a lack of transparency regarding timekeeping [61, 62].

Summing up, the analysis of the institutional design of the public hospital sector with regard to special-fee payment and dual practice, in combination with the knowledge from the relevant scientific literature and the sparsely available Austrian-specific studies, has revealed that there are several gateways to institutional corruption in Austria. Table 4 summarizes potential conduct as a result of legal regulations that has the potential to put the achievement of the purposes of ‘good health,’ ‘equity’ and ‘efficiency’ at risk.

**Table 4:** Potential gateways to institutional corruption

Breakdown/motivation structure according to federal/state laws	potentially promotes	puts at risk
• Existence of a physician fee per se	prioritization of supplementary- insured patients, increasing/by-passing public waiting lists	equal treatment for equal needs in terms of volume, range, timing and access
• Restriction of hospital and physician fees to inpatient (day) care	inpatient care of outpatient (ambulatory) care	service provision at the ‘best point of service’ and relief of the inpatient sector
• Limiting the special-class bed capacity to 25% of the overall bed capacity	high capacity utilization	reasonable reduction in the overall bed capacity and capacity-reducing innovations
• Tariffication of physician fees	overprovision of medical services, prolongation of length of stay	equal and efficient service provision
• Possibility of dual practice	redirection of profitable patients to private practice/private hospital	service provision at the ‘best point of service,’ coordinated service provision, prevention and decrease of parallel structures and relief of the inpatient sector
	overprovision of medical services through self-referral	
	focus on private practice at the expense of public health care provision	
	misuse of public resources for privately offered medical services	
	absenteeism	
	outflow of public hospitals’ resources to private hospitals	

## Conclusions

The aim of this paper was to investigate whether the Austrian health care system is prone to institutional corruption and, if so, through which channels institutional corruption has developed. Institutional corruption may arise as a consequence of the emergence of (financial) conflicts of interest that result from the institutional design and that enforce the tension between physicians’ fiduciary task of acting in the best interests of the patient and their financial interests. Medical decisions that are made in favor of physicians’ financial benefit usually do not harm patients directly, as their effects are rather indirect: inefficient resource use as a consequence of supplier-induced demand (concerning the volume, range and frequency of services), inequity as a consequence of a two-tier medicine system (concerning the range, timing and continuity of and access to medical services) and, subsequently, impairment of the population’s good health if the financial viability of the overall health care system suffers and equal access for equal needs cannot be guaranteed.

The paper focused on special fees in the form of physician fees, because they represent an important source of additional income for employed public hospital physicians. Physician fees are paid for treating supplementary-insured patients in the special class of public hospitals. The impact of dual practice was also taken into consideration, because dual practice as self-employed physician or attending physician in private hospitals opens up a broad spectrum of opportunities for employed hospital physicians to act in an entrepreneurial manner, which, in turn, is supposed to interact with the service provision provided in the special class of the public hospital.

The institutional design, which regulates special fees and dual practice in the public hospital sector in Austria, has grown historically in an attempt to contribute to the financing of the public health sector and to commit highly qualified physicians to the public hospital sector. The rule-making resulted in extremely complex and further proliferating amendments to the respective regulations. The institutional design, however, has been put into place without clearly and comprehensively addressing and assessing the overall consequences for the purposes of the health care system as can be seen by the potential gateways to institutional corruption which have been identified for a single Austrian state. The results showed that there are in fact legal provisions and directives, which have the potential to promote behavior on the part of the employed public hospital physician that systematically undermines the achievement of the Austrian public health system’s primary purposes of ‘good health,’ ‘equity’ and ‘efficiency.’ This corresponds to the definition of ‘institutional corruption’ in the relevant literature. In particular, linking the additional income of public hospital physicians in terms of physician fees to inpatient capacity utilization and paying fees per intervention and/or day bears the risk of inefficient service provision as a result of potential supplier-induced demand and overprovision of services. Restricting the payment of special fees to inpatient care and authorizing dual practice, especially in private hospitals, puts at risk the service provision at the best point of service and the relief of the cost-intensive inpatient sector. Paying physician fees to employed hospital physicians at all may lead to two-tier medicine in the form of prioritizing the supplementary-insured patients and/or increasing or by-passing public waiting lists. Following some rare Austria-specific studies and general findings in the relevant scientific literature, the emergence and existence, respectively, of two-tier medicine seem likely.

While the prevailing contribution identified only potential gateways to institutional corruption following the lack of access to empirical data and missing data, future research needs to concentrate on the actual effects on the medical decisions of public hospital physicians caused by the conflicts of interest resulting from the institutional design regulating physician fees and dual practice. The hypotheses, which were derived on the basis of the rarely available data for Austria and the general findings in the relevant literature, clearly need to be tested empirically. In other words, a reliable and accessible database covering relevant information regarding, for example, the revenues/income from special fees at the hospital and the physician level, and across medical disciplines, the type and extent of dual practice of public hospital physicians, the service provision in terms of the volume, range and timing of services inside and outside the hospital, the referral and timing of referrals and the waiting times for medical treatment, each disaggregated by the insurance status of the patient, is required to assess reliably the actual effects of the current institutional design on the ascribed purposes of the Austrian public health care system.

Empirical evidence regarding the actual effects is a prerequisite for the development of reform proposals in terms of conflict regulation, should the hypotheses regarding institutional corruption prove to be true. If there is still commitment to the current primary purposes of ‘good health,’ ‘equity’ and ‘efficiency,’ possible options for reform range from reshaping the payment system of public hospital physicians towards higher salaries as compensation for variable income components (physician fees) to changing the tariffication and/or allocation of physician fees and to reregulating the options regarding the dual practice of employed public hospital physicians, particularly in private hospitals. It might, however, also become necessary to reregulate the service provision of self-employed physicians and the organization of SHI: starting points range from revising the organization and financing of primary health care to overthinking the overall number of contracts offered in the present system and to uniting the SHI funds (or even all the social security funds) to avoid differences in the accounting systems and in the benefits and contributions between the socially insured. If, however, there is no longer commitment to the purposes of ‘good health,’ ‘equity’ and ‘efficiency’ or the purposes can no longer be maintained, further options may emerge. Final considerations and recommendations can only be made if the nature and extent of the alleged negative effects of physician fees and dual practice on the primary purposes of the Austrian health care system are determined comprehensively.
